# Association between *APOC1* Polymorphism and Alzheimer’s Disease: A Case-Control Study and Meta-Analysis

**DOI:** 10.1371/journal.pone.0087017

**Published:** 2014-01-31

**Authors:** Qin Zhou, Fan Zhao, Ze-ping Lv, Chen-guang Zheng, Wei-dong Zheng, Liang Sun, Na-na Wang, Shenghang Pang, Fabiana Michelsen de Andrade, Mian Fu, Xiang-hua He, Juan Hui, Wen-yu Jiang, Chu-yu Yang, Xiao-hong Shi, Xiao-quan Zhu, Guo-fang Pang, Yi-ge Yang, Hai-qun Xie, Wan-dong Zhang, Cai-you Hu, Ze Yang

**Affiliations:** 1 The Key Laboratory of Geriatrics, Beijing Hospital & Beijing Institute of Geriatrics, Ministry of Health, Beijing, China; 2 Department of Neurology, Jiangbin hospital, Guangxi Zhuang Autonomous Region, Nanning, China; 3 Health Science Institute, Universidade Feevale, Novo Hamburgo, Rio Grande do Sul, Brazil; 4 Institutes for Biological Sciences, National Research Council of Canada, Ottawa, Canada; San Francisco Coordinating Center, United States of America

## Abstract

**Background:**

Previous association studies examining the relationship between the *APOC1* polymorphism and susceptibility to Alzheimer’s disease (AD) have shown conflicting results, and it is not clear if an *APOC1* variant acts as a genetic risk factor in AD etiology across multiple populations.

**Methods:**

To confirm the risk association between *APOC1* and AD, we designed a case-control study and also performed a meta-analysis of previously published studies.

**Results:**

Seventy-nine patients with AD and one hundred fifty-six unrelated controls were included in case-control study. No association was found between the variation of *APOC1* and AD in stage 1 of our study. However, our meta-analysis pooled a total of 2092 AD patients and 2685 controls. The *APOC1* rs11568822 polymorphism was associated with increased AD risk in Caucasians, Asians and Caribbean Hispanics, but not in African Americans. *APOE* ε4 carriers harboring the *APOC1* insertion allele, were more prevalent in AD patients than controls (χ^2^ = 119.46, OR = 2.79, 95% CI = 2.31–3.36, *P*<0.01).

**Conclusions:**

The *APOC1* insertion allele, in combination with *APOE* ε4, likely serves as a potential risk factor for developing AD.

## Introduction

Alzheimer’s disease (AD) is a neurodegenerative disease and the most common cause of dementia in the elderly, with the significant clinical manifestation of slow but progressive loss of cognitive function, especially memory dysfunction. 20–30 million people worldwide suffer from this devastating disease [1]. The diagnosis of sporadic Alzheimer’s disease (SAD) refers to patients with AD, usually onset after age 65, accounting for about 95% of AD cases [2]. AD belongs to diseases with a complex pathogenesis, mainly attributing to genetic and environmental risk factors, and the interactions of multiple genetic and environmental factors probably play an important role in both development and progression of AD. Twin studies have indicated that AD is a highly heritable disease, with heritability estimates 60% to 80% [3]. To date, the allele ε4 of apolipoprotein E (*APOE*) is the only unequivocally genetic risk factor for SAD [4]. Beyond *APOE*, 695 AD candidate genes have already been investigated in case-control studies to explore their association with AD, but their role is not definitely established [5].

Apolipoprotein C-I (*APOC1*) is one of the AD candidate genes, whose variance such as rs11568822 associated with the AD risk that might have a functional relation to AD pathophysiology. The key pathologic characteristics of AD are the generation of neuritic plaques by extracellular deposition of amyloid β-peptide (Αβ), intracellular formation of neurofibrillary tangles and neuronal loss [6]. Apolipoprotein C1 (ApoC1) encoded by *APOC1* is a member of apolipoprotein family. ApoC1 in partnership with apoE participate in a wide variety of biological processes of cholesterol metabolism, membrane remodeling, neuronal apoptosis and reorganization [7]. The growing evidence from clinical and pathological studies indicates the presence of important relationships between the ongoing deterioration of brain cholesterol metabolic disturbance and the AD pathophysiology [7], [8]. Thus, *APOC1* variants might be involved with pathological mechanism of AD.

A 4-bp CGTT insertion/deletion polymorphism of *APOC1* gene (rs11568822) has been identified in the promoter region [9], [10]. Although it modifies the encoded protein indirectly, polymorphism in the promoter region may potentially regulate the gene expression and therefore influences susceptibility to AD. However, it is not neglected fact that *APOC1* is located adjacent to *APOE* at the long arm of chromosome 19 and constructs a cluster with other apolipoprotein gene. The study by Bertram *et al.* suggested that *APOC1* rs11568822 polymorphism associated with AD risk could be related to linkage disequilibrium with *APOE* because of synteny and collinearity in chromosome 19 [11]. But so far, it hasn’t actually ever been proved if *APOC1* rs11568822 polymorphism is associated with AD risk, and there is a lack of the meta-analysis evidences from different ethnic groups from multiple countries in the world. Although the association between the *APOC1* rs11568822 polymorphism and AD risk has been hotly debated in many studies [9], [10], [12], [13], [14], [15], [16], [17], [18], [19], [20], [21], [22], [23], the results of these studies are conflicting and ambiguous, partially due to the relatively small sample size of individual studies and a wide range of ethnic groups. Therefore, we conducted a two-stage study to obtain more comprehensive understanding of the relationship between *APOC1* rs11568822 polymorphism and AD risk in a wide range of populations. In stage 1, we conducted a case-control association study using the sample recruited from south China. In stage 2, we performed a meta-analysis to pool all published case-control studies to test this polymorphism association with AD, to examine it by stratification according to *APOE* ε4 status and to analyze its accumulation effect with *APOE* ε4.

## Materials and Methods

### 2.1 Stage 1: Case-control Association Study

#### 2.1.1 Patient sample and controls

Seventy-nine patients with AD were recruited from inpatients and outpatients sections of Department of Neurology, Jiangbin Hospital in Guangxi Zhuang Autonomous Region, China. Two trained neurologists completed all the assessments together by interviewing patients and their reliable caregivers. All the patients included met the National Institute of Neurological Disorders and Stroke-Alzheimer Diseases and Related Disorders Association (NINCDS-ADRDA) criteria [24] for probable AD, with a Hachinski Ischemia Scale score [25] less than 4. All patients had no family history of AD. The onset age of patients were at 65 years or older. In addition, each patient underwent a complete examination including physical examination, laboratory testing and brain MRI to exclude causes of dementia other than AD, other nervous system diseases and psychiatric diseases.

One hundred fifty-six unrelated controls were randomly selected from healthy elderly people carrying the physical examination in Jiangbin Hospital, aged 65 years or older. The controls had normal physical examination and a mini-mental state examination (MMSE) score more than 28, without family history of AD, other nervous system diseases and psychiatric diseases.

The study adhered to the principles of the Declaration of Helsinki. Approval for this study was obtained from the Jiangbin Hospital Ethical Committee. Written informed consent was obtained from all participants or their guardians before study participation.

#### 2.1.2 Laboratory methods

Blood samples of each included participants were collected by standard venipuncture into evacuated vacuum tubes with ethylene diaminetetraacetic acid (EDTA). Genomic DNA was extracted from whole blood samples using standard DNA isolation methods [26].

Genotyping for *APOC1* (rs11568822) was carried out by using polymerase chain reaction–restriction fragment length polymorphism (PCR–RFLP) methods. The forward and reverse primers used for *APOC1* were 5′-tttgagctcggctcttgagacaggaa-3′ and 5′-ggtcccgggcacttcccttagcccca-3′. PCR reaction system (total volume 20 μL) to detect *APOC1* gene contained 800 μmol/L dNTP mixtures (200 μmol/L each dNTP), forward and reverse primers (each 5 pmol), 2.0 μL 10×buffer, 50 ng template DNA, and 1 U Taq DNA polymerase. The thermal cycle profile for *APOC1* were as follows: initial denaturation at 94°C for 5 minutes; 35 cycles of 94°C for 35 seconds, 61°C for 35 seconds, and 72°C for 35 seconds; 72°C for 5 minutes. The PCR products were identified by 8% non-denaturing polyacrylamide gel electrophoresis. After identified by electrophoresis, the PCR products were subsequently digested in a total volume 20 μL HpaI restriction endonuclease reaction system at 37°C for 4 hours. The digestion products were visualized and autoradiographed after being separated by non-denaturing polyacrylamide gel electrophoresis. Digestion of the PCR products yielded bands of 222 bp in del/del genotype, 66 and 160 bp in ins/ins genotype, all 3 bands in ins/del genotype (**Figure S1**) and were confirmed by sequencing.

### 2.2 Stage 2: Meta-analysis

#### 2.2.1 Search strategy

Potential eligible studies were identified by electronic searches from the Medline, EMBSE, the Cochrane Library, and Chinese Biomedical Literature Database (CBM) up to 24 August 2013. “Alzheimer Disease” and “Apolipoprotein C-I” were used as the MeSH terms to perform the subject searching. “Alzheimer Disease” and (Apolipoprotein C-I or Apolipoprotein CI or APOC1 or APOCI or ApoC-I or apo-CIB or rs11568822) were used as the text words to perform the text word searching. An internet database about AD (AlzGene, updated 18 April 2011) was searched using APOC1 as the search term in 24 August 2013. We also reviewed the reference lists of retrieved articles to check for additional reports of relevant studies. There were no language restrictions.

#### 2.2.2 Study selection and data extraction

Studies were included if they met all of the following criteria: (1) the study should evaluate polymorphism of *APOC1* rs11568822 and SAD risk; (2) case-control studies were based on human; (3) sufficient data (the sample size, allele frequencies, genotype, or other useful information) were available; (4) the most complete results were used in the case of multiple publications from the same study.

The following information was collected by two independent reviewers using a predetermined data collection form: the first author’s name, year of publication, country of origin, ethnicity, type of AD, AD diagnosis criteria, source of controls, method of genotyping, total number of AD and controls, age of AD and control group, proportion of male in AD and control group, and numbers of AD and control group with different genotypes. Original articles reported the results on different subpopulations were treated as separate studies. We tried to contact with the original investigators to get the missing data of included studies. Two reviewers independently identified potential relevant studies and collected the useful information based on predetermined strategies. Disagreements were resolved by discussion. If disagreements persisted, the third review author arbitrated.

#### 2.2.3 Statistical analysis

In stage 1, statistical analyses were performed using SPSS statistical software, version 16.0 (Chicago, USA). Age was analysed by Student’s t-test. Hardy-Weinberg equilibrium (HWE) in control group, gender distributions, allele and genotypes between groups were analysed by Pearson χ^2^ test. Two-locus linkage disequilibrium between *APOC1* and *APOE* were examined using SHEsis software platform, which is available online [27]. All tests were two-sided. The significance level was set at *P*≤0.05.

In stage 2, statistical analyses were performed using Comprehensive Meta Analysis version 2.2.064 (U.S. and the U.K.) and Review Manager 5.2 (Copenhagen, Denmark). The strength of association between *APOC1* rs11568822 polymorphism and AD risk was measured by crude odds ratios (ORs) and 95% confidence intervals (CIs). The pooled ORs were calculated for allelic comparison (ins vs. del), recessive (ins/ins vs. ins/del+del/del), dominant (ins/ins+ins/del vs. del/del), overdominant (ins/del vs. ins/ins+del/del), homozygote comparison (ins/ins vs. del/del), and heterozygote comparison models (ins/del vs. del/del). And the pooled ORs were also calculated for the comparison between insertion homozygote and heterozygote (ins/ins vs. ins/del). Statistical heterogeneity among the studies was evaluated using a Chi^2^ test and the I^2^ statistic. If a *P_h_* value for heterogeneity was less than or equal to 0.05 in Chi^2^ test and I^2^ statistic was more than or equal to 50% (*P_h_*≤0.05 and I^2^≥50%), it indicated substantial heterogeneity between included studies [24]. Random effects model was chosen to perform meta-analysis. If a *P_h_* value for heterogeneity was more than 0.05 in Chi^2^ test or I^2^ statistic was less than 50% (*P_h_*>0.05 or I^2^<50%), it indicated no substantial heterogeneity between included studies. Fix effects model was chosen to perform meta-analysis. Subgroup analysis was performed by different ethnic groups to evaluate the ethnic effects. Sensitivity analysis was performed to test the robustness of the results. First, all the meta-analysis was performed twice by using different analyzed model. For example, if random effects model was chosen at first, we used fix effects model to repeat the analysis to test the influence of different analysed model. Second, all the analyses were repeated by sequence excluding each individual study to test the influence of each study. Third, all the analyses were also repeated by sequence excluding all studies without original data on genotypes at a time to test the influence of those studies. Publication bias was determined by visual inspection of the funnel plot and Egger's linear regression test. A funnel plot is a simple scatter plot whose shape is available to estimate publication bias. In the absence of bias the plot should approximately resemble a symmetrical inverted funnel. Population attributable risk (PAR) was estimated for both *APOC1* and *APOE* by the equation mentioned in Zheng *et al.* [28].

## Results

### 3.1 Stage 1: Case-control Association Study

For AD patients, the average age at examination was 72.8±9.5 years and 50.5% were male. Meanwhile, the average age of controls was 71.2±9.3 years and 59.0% were male. Age and gender distribution were comparable in both groups. Genotype distributions of *APOC1* polymorphism in controls was in HWE. No associations were found in four genetic models (allelic comparison, dominant, overdominant, or heterozygote comparison models) (**Table 1**). When stratification according to *APOE* status, there were also no associations found either in *APOE* ε4 carriers or non-carriers using those four genetic models (allelic comparison, dominant, overdominant, or heterozygote comparison models). Only four AD patients and one control were ins/ins genotype carriers in our study. Analysis wasn’t performed in the rest three models (recessive, homozygote comparison models, and the comparison between insertion homozygote and heterozygote), because the number of ins/ins genotype carriers was too small to interpret. Positive linkage disequilibrium between *APOC1* and *APOE* was observed both in AD (D’ = 0.859, r^2^ = 0.475) and control group (D’ = 0.752, r^2^ = 0.497). The deletion allele of *APOC1* and *APOE* ε4 were found in positive linkage disequilibrium (χ^2^ = 7.12, P<0.01).

**Table 1:** *APOC1* (rs11568822) polymorphism and AD.

Genetic models	AD	controls	χ^2^	OR	95% CI	*P*
Allelic comparison model						
ins	27	48				
del	131	264	0.23	1.13	0.68–1.90	0.63
Dominant model						
ins/ins+ins/del	23	47				
del/del	56	109	0.03	0.95	0.53–1.73	0.87
Overdominant model						
ins/del	19	46				
ins/ins+del/del	60	110	0.78	0.76	0.41–1.41	0.38
Heterozygote comparison model						
ins/del	19	46				
del/del	56	109	0.47	0.80	0.43–1.50	0.49

Abbreviations: AD, Alzheimer’s disease; OR, odds ratios; CI, confidence interval; ins, insertion; del, deletion.

### 3.2 Stage 2: Meta-analysis

#### 3.2.1 Study inclusion and characteristics

**Figure 1:**
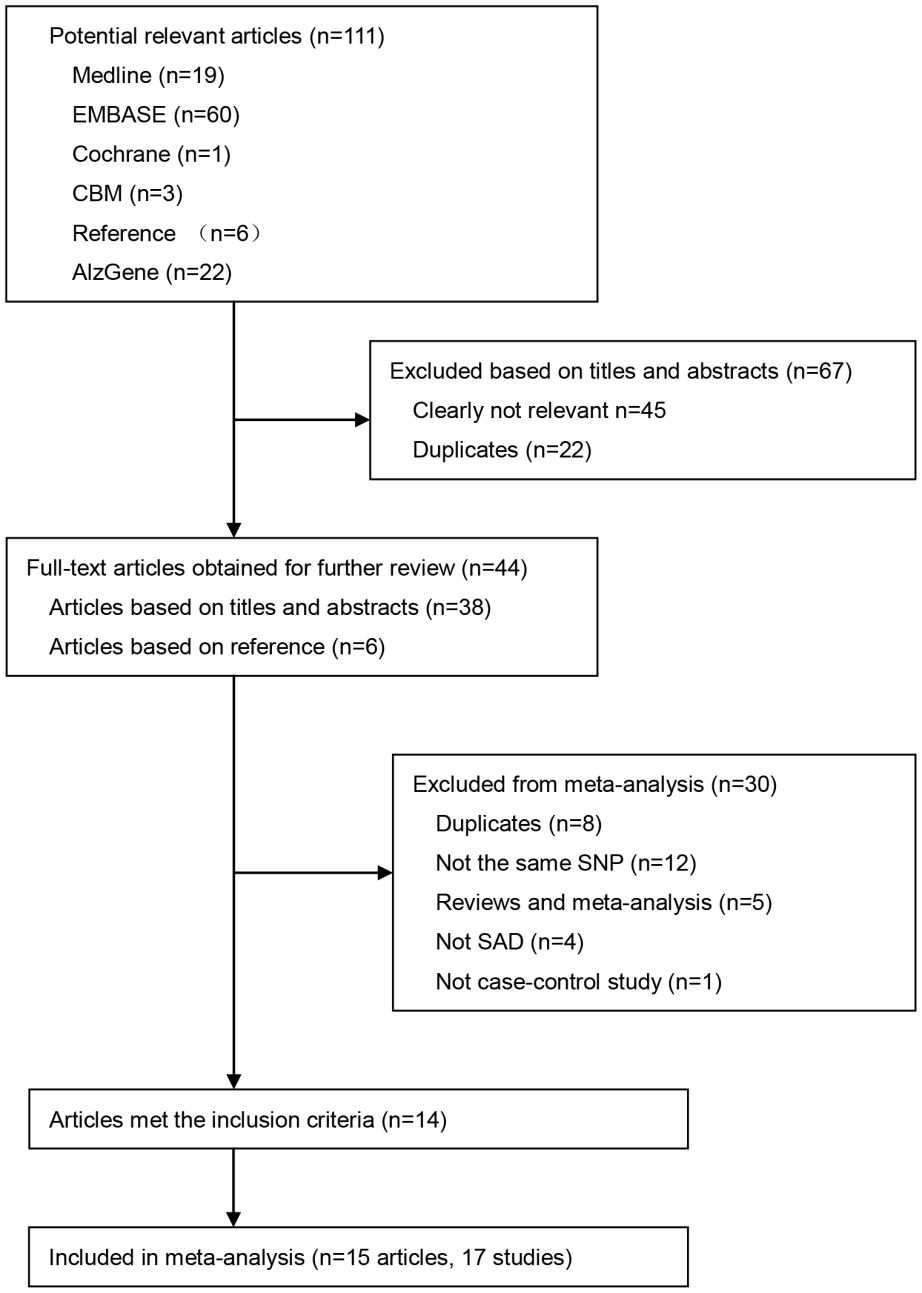
Flow diagram of study selection. A total of 15 articles, 14 published articles plus our study, were included in the meta-analysis. Of them, two published articles were regarded as two different studies as they included different subpopulations.

For the meta-analysis, our initial search using predetermined search strategies identified 111 articles. After exclusion of 67 records because of lack of relevance or duplication, 44 full-text articles were retrieved for eligibility assessment. Of the 44 full-text articles, 30 were further excluded for various reasons (**Figure 1**). Fourteen case-control articles met the preliminary inclusion criteria [9], [10], [12], [13], [14], [15], [16], [17], [18], [19], [20], [21], [22], [23]. The study by Chartier-Harlin *et al*. [15] was regarded as two distinct studies as it included two different AD sub-diagnoses. Similarly, Tycko *et al*. [12] was also regarded as two studies as it included two different ethnic groups (**Table 2**). Thus, excluding our study, there were 14 published articles (16 studies) included in the meta-analysis (**Figure 1**). There were no overlapping participants in these studies.

**Table 2:** Main characteristics of included studies in meta-analysis.

Studyname	Country	Ethnicgroups	Type of AD	Diagnostic criteria	Source of controls	HWE	AD			Controls		
							N	Age	Male (%)	N	Included age	Male (%)
Chartier-Harlin-France1994	France	Caucasian	SLOAD	DSM-III-R	Matched environment	B	36	78.0±9.0^▴^	NI	38	80.0±8.0	NI
Chartier-Harlin-U.K.1994	UK	Caucasian	SEOAD	NI	Random sample in UK	B	34	57.0±5.0^▴^	NI	36	55.0±7.5	NI
Drigalenko 1998	NI	Caucasian	SLOAD	NINCDS-ADRDA	Spouses (53); Matched ethnicity and environment (173)	NI	176	≥65 years^▴^	34.1	226	NI	43.8
Lucatelli 2011	Brazil	Caucasian	SAD	MMSE and DSM-IV	>60 years general population	NI	35	74.7±4.1^△^	NI	85	68.3±6.0	NI
Mullan 1996	USA	Caucasian	SAD	MMSE and NINCDS-ADRDA	Residents in retirement communities	NI	214	73.0±8.0^▴^	NI	496	74.0±8.0	NI
Petit-Turcotte 2001	Canada	Caucasian	SAD	Neuropathology	No neuropathological changes relevant to AD or other neurodegenerative disorder	NI	142	NI	NI	67	NI	NI
Poduslo 1998	USA	Caucasian	SAD	NINCDS-ADRDA; Neuropathology (50)	Spouses and siblings of matched ethnicity and environment	NI	246	69.9±8.5^▴^	NI	289	72.6±8.2	41.5
Retz 2001	Germany	Caucasian	SAD	NINCDS-ADRDA and ICD-10	Psychiatric patients	NI	63	66.6±11.9	31.7	162	70.6±8.2	38.2
Scacchi 1999	Italy	Caucasian	SLOAD	NINCDS-ADRDA and DSM-III-R	Matched sex and age	B	85	86.1±3.8^△^	23.5	156	83.8±3.2	41.7
Chuang 2010	China	Asian	SAD	NINCDS-ADRDA and DSM-IV	Volunteers matched ethnicity and environment (some were spouses)	B	127	NI	37.1	191	NI	NI
Kamino 1996	Japan	Asian	SAD	NINCDS-ADRDA	Matched age	NI	99	75.4±7.0^▴^	NI	52	88.2±5.38	NI
Ki 2002	Korea	Asian	SLOAD	NINCDS-ADRDA	Healthy volunteers matched age	B	120	69.7±6.2^▴^	24.2	132	70.2±7.1	24.2
Shi 2004	China	Asian	SAD	NINCDS-ADRDA	Matched age	B	257	73.3±8.3^▴^	40.5	242	80.0±7.6	47.1
Yang 2003	China	Asian	SAD	NINCDS-ADRDA	Recruited from hospital	NI	183	74.6^△^	41.5	133	NI	51.9
Zhou 2013*	China	Asian	SLOAD	NINCDS-ADRDA	Matched age and gender	B	79	72. 8±9. 5^△^	50.6	156	71. 2±9. 3	59.0
Tycko-African 2004	USA	African American	SAD	NINCDS-ADRDA and CDR≥1	Random sample of elderly people	B	142	82.3^▴^	NI	251	78.2	NI
Tycko-Hispanic 2004	USA	Caribbean Hispanic	SAD	NINCDS-ADRDA and CDR≥1	Random sample of elderly people	B	230	81.9^▴^	NI	359	77.5	NI
Tycko 2004	USA	African American and Hispanic	SAD	NINCDS-ADRDA and CDR≥1	Random sample of elderly people	B	372	79.5±6.6^△^	27.9	610	NI	27.9

Abbreviations: NI, no information; AD, Alzheimer's disease; SLOAD, sporadic late-onset Alzheimer's disease; SEAD, sporadic early-onset Alzheimer's disease; SAD, sporadic Alzheimer's disease; DSM, Diagnostic and Statistical Manual of Mental Disorders; * Our study; ^▴^Age of onset; ^△^Included age.

NINCDS-ADRDA, the National Institute of Neurological Disorders and Stoke–Alzheimer Diseases and Related Disorders Association; MMSE, mini mental state examination; ICD, International Classification of diseases; CDR, clinical dementia rating; HWE,Hardy-Weinberg equilibrium; B, balance; N, number.

The detailed characteristics of these studies were presented in **Table 2**. Participants were recruited from Europe, North America, East Asia and Latin America. In total, 2092 cases and 2685 controls were included, consisting of 2115 Caucasians, 1771 Asians, 531 Caribbean Hispanics and 360 African Americans. All patients were diagnosed with SAD, specifically, either sporadic late-onset Alzheimer’s disease (SLOAD) [10], [15], [20], [21] or sporadic early-onset Alzheimer’s disease (SEOAD) [15]. Overall, neuropathology was only used as the AD diagnosis criteria by Petit-Turcotte *et al*. [13] and partially by Poduslo *et al*. [23]. The most common AD diagnostic criteria was from NINCDS-ADRDA [10], [12], [14], [16], [17], [18], [19], [20], [21], [22], [23]. Four studies [9], [15], [17], [21] used the Diagnostic and Statistical Manual of Mental Disorders, third/fourth edition (DSM-III-R or DSM-IV) criteria, and one study [22] used the International Classification of diseases, Tenth Revision (ICD-10). The source of controls varied (**Table 2**). Few studies had ethnic and environment matched controls. Only half the studies stated that in controls, the *APOC1* polymorphism genotype distribution was in HWE [10], [12], [15], [16], [17], [21]. All the studies used PCR–RFLP methods to genotype the *APOC1* rs11568822 polymorphism.

#### 3.2.2 Quantitative synthesis

**Figure 2:**
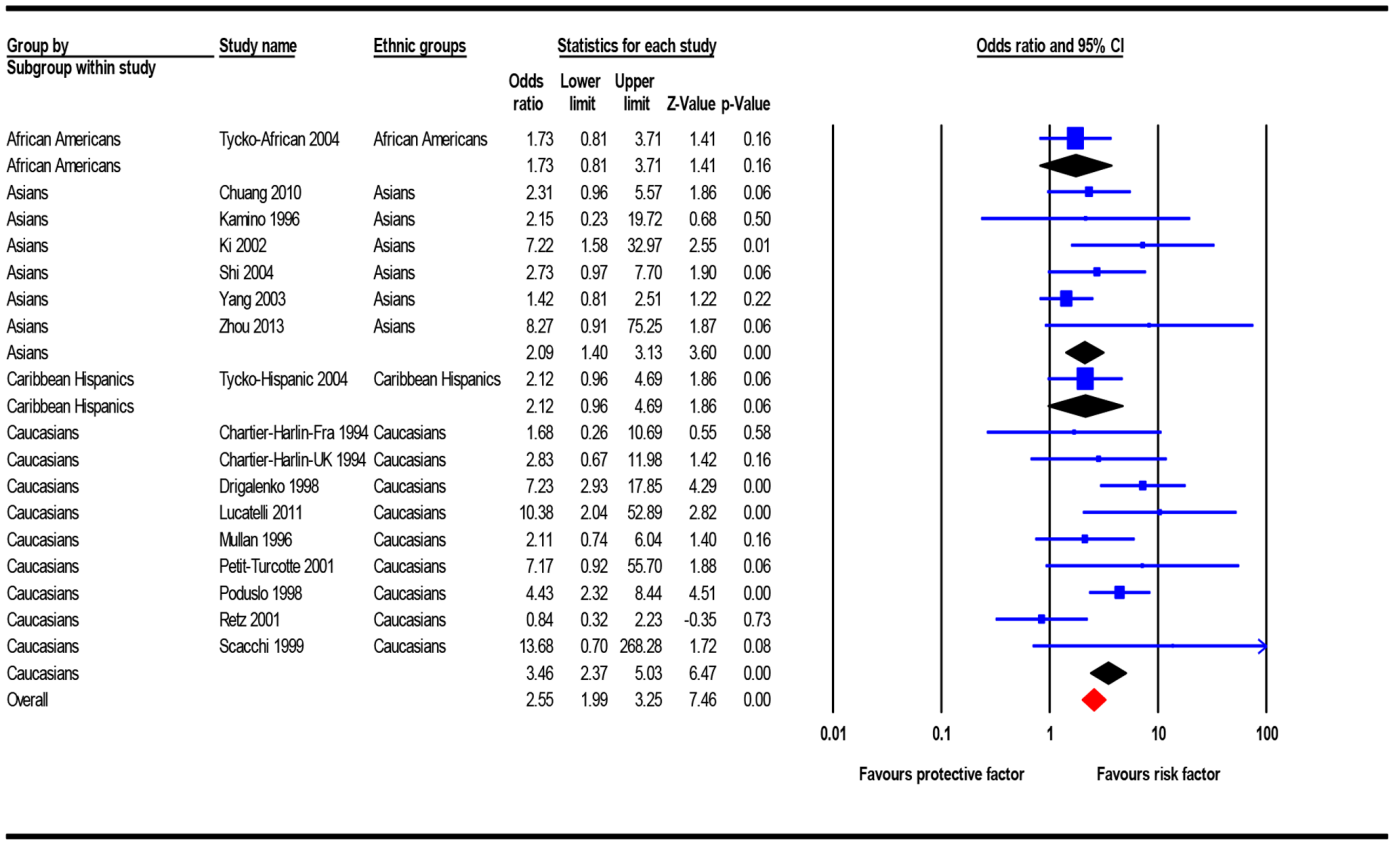
Forest plot for the association between the *APOC1* rs11568822 polymorphism and AD risk using recessive model (fix effects model).

The results of meta-analysis were presented in detail in **Table 3**. For allelic comparison, and recessive, dominant and overdominant models, 2092 AD patients and 2685 controls were included. Allelic comparison found the *APOC1* insertion allele was more prevalent in AD patients than controls (heterogeneity: *P_h_*<0.001, *I^2^* = 71.36%; OR = 1.84, 95%CI = 1.34–2.52). Association between *APOC1* variant genotypes and increased AD risk was observed using the recessive (heterogeneity: *P_h_* = 0.05, *I^2^* = 39.68%; OR = 2.55, 95%CI = 1.99–3.25) (**Figure 2**), dominant (heterogeneity: *P_h_*<0.001, *I^2^* = 71.56%; OR = 1.96, 95%CI = 1.28–3.02), and overdominant (heterogeneity: *P_h_* = 0.002, *I^2^* = 57.59%; OR = 1.57, 95%CI = 1.12–2.22) models. Using the homozygote comparison model, 1198 AD patients and 1876 controls were included, and showed that the ins/ins genotype was more prevalent in AD patients, compared with the del/del genotype (heterogeneity: *P_h_* = 0.01, *I^2^* = 52.60%; OR = 3.40, 95%CI = 1.88–6.15). Using the heterozygote comparison model, 1855 AD patients and 2565 controls were included, and showed that the ins/del genotype was more prevalent in AD patients, compared with the del/del genotype (heterogeneity: *P_h_*<0.001, *I^2^* = 66.43%; OR = 1.78, 95%CI = 1.17–2.72). To compare insertion homozygote and heterozygote, 1131 AD patients and 929 controls were included. The results showed that the ins/ins genotype was more prevalent in AD patients, compared with the ins/del genotype (heterogeneity: *P_h_* = 0.31, *I^2^* = 12.64%; OR = 1.79, 95%CI = 1.38–2.31).

**Table 3:** Summary of meta-analysis results in different models.

Genetic models	Ethnic groups	N	Groups		Heterogeneity		Mode	OR	95% CI	
			AD	controls	*P*_h_	I^2^ (%)			Lower limit	Upper limit
Allelic comparison model	**Caucasians**	9	904	1211	0.09	42.36	F	**2.49**	**2.16**	**2.87**
(ins vs. del)	**Asians**	6	865	906	0.002	73.31	R	**1.74**	**1.32**	**2.30**
	**Caribbean Hispanics**	1	193	338	1.00	0.00	F	**1.72**	**1.24**	**2.38**
	African Americans	1	130	230	1.00	0.00	F	1.22	0.86	1.72
	**Total**	17	2092	2685	<0.001	71.36	R	**1.84**	**1.34**	**2.52**
Recessive model	**Caucasians**	9	904	1211	0.05	49.26	F	**3.46**	**2.37**	**5.03**
(ins/insvs.ins/del+del/del)	**Sensitivity-C**	5	442	779	0.01	71.10	R	**3.39**	**1.47**	**7.78**
	**Asians**	6	865	906	0.30	18.23	F	**2.09**	**1.40**	**3.13**
	**Sensitivity-A**	5	682	773	0.64	0.00	F	**3.09**	**1.75**	**5.48**
	Caribbean Hispanics	1	193	338	1.00	0.00	F	2.13	0.96	4.69
	African Americans	1	130	230	1.00	0.00	F	1.73	0.81	3.71
	**Total**	17	2092	2685	0.05	39.68	F	**2.55**	**1.99**	**3.25**
	**Sensitivity-T**	12	1447	2120	0.07	40.35	F	**2.62**	**1.91**	**3.60**
Dominant model	**Caucasians**	9	904	1211	0.41	2.86	F	**3.12**	**2.59**	**3.77**
(ins/ins+ins/del vs. del/del)	**Sensitivity-C**	5	442	779	0.60	0.00	F	**2.87**	**2.24**	**3.68**
	**Asians**	6	865	906	0.001	74.80	R	**1.85**	**1.34**	**2.56**
	**Sensitivity-A**	5	682	773	0.001	78.80	R	**1.99**	**1.34**	**2.95**
	**Caribbean Hispanics**	1	193	338	1.00	0.00	F	**1.78**	**1.21**	**2.61**
	African Americans	1	130	230	1.00	0.00	F	1.14	0.73	1.76
	**Total**	17	2092	2685	<0.001	71.56	R	**1.96**	**1.28**	**3.02**
	**Sensitivity-T**	12	1447	2120	<0.001	70.35	R	**2.00**	**1.33**	**3.02**
Overdominant model	**Caucasians**	9	904	1211	0.86	0.00	F	**2.13**	**1.77**	**2.56**
(ins/del vs ins/ins+del/del)	**Sensitivity-C**	5	442	779	0.97	0.00	F	**2.07**	**1.62**	**2.64**
	**Asians**	6	865	906	0.002	73.44	R	**1.49**	**1.12**	**1.99**
	**Sensitivity-A**	5	682	773	0.002	73.45	R	**1.64**	**1.14**	**2.35**
	**Caribbean Hispanics**	1	193	338	1.00	0.00	F	**1.56**	**1.04**	**2.35**
	African Americans	1	130	230	1.00	0.00	F	0.95	0.60	1.50
	**Total**	17	2092	2685	0.002	57.59	R	**1.57**	**1.12**	**2.22**
	**Sensitivity-T**	12	1447	2120	0.01	58.69	R	**1.63**	**1.15**	**2.30**
Homozygote comparison model	**Caucasians**	9	461	829	0.04	51.26	R	**5.46**	**3.12**	**9.58**
(ins/ins vs. del/del)	**Sensitivity-C**	5	224	533	0.01	70.18	R	**5.38**	**2.31**	**12.53**
	**Asians**	6	512	626	0.30	17.34	F	**2.66**	**1.73**	**4.10**
	**Sensitivity-A**	5	420	557	0.64	0.00	F	**3.85**	**2.16**	**6.88**
	**Caribbean Hispanics**	1	137	268	1.00	0.00	F	**2.43**	**1.09**	**5.41**
	African Americans	1	88	153	1.00	0.00	F	1.74	0.80	3.80
	**Total**	17	1061	1608	0.01	52.60	R	**3.40**	**1.88**	**6.15**
	**Sensitivity-T**	12	869	1511	0.03	48.78	F	**3.41**	**2.47**	**4.71**
Heterozygote comparison model	**Caucasians**	9	784	1159	0.78	0.00	F	**2.78**	**2.29**	**3.38**
(ins/del vs. del/del)	**Sensitivity-C**	5	389	746	0.95	0.00	F	**2.60**	**2.01**	**3.36**
	**Asians**	6	776	865	0.002	73.89	R	**1.69**	**1.24**	**2.30**
	**Sensitivity-A**	5	635	755	0.001	78.27	R	**1.81**	**1.23**	**2.66**
	**Caribbean Hispanics**	1	179	326	1.00	0.00	F	**1.67**	**1.10**	**2.51**
	African Americans	1	116	215	1.00	0.00	F	1.02	0.64	1.63
	**Total**	17	1676	2239	<0.001	66.43	R	**1.78**	**1.17**	**2.72**
	**Sensitivity-T**	12	1140	1716	0.001	66.57	R	**1.82**	**1.21**	**2.75**
Homozygote vs. heterozygote	**Caucasians**	9	563	434	0.12	37.25	F	**2.04**	**1.38**	**3.01**
(ins/ins vs. ins/del)	Sensitivity-C	5	271	279	0.02	67.01	R	2.01	0.87	4.65
	**Asians**	6	442	321	0.45	0.00	F	**1.63**	**1.07**	**2.49**
	**Sensitivity-A**	5	309	234	0.48	0.00	F	**2.06**	**1.14**	**3.74**
	Caribbean Hispanics	1	70	82	1.00	0.00	F	1.46	0.62	3.40
	African Americans	1	56	92	1.00	0.00	F	1.71	0.75	3.88
	**Total**	17	1061	929	0.31	12.64	F	**1.79**	**1.38**	**2.31**
	**Sensitivity-T**	12	636	605	0.14	31.64	F	**1.83**	**1.31**	**2.54**

Abbreviations: N, number; AD, Alzheimer's disease; *P*_h_, *P* value for heterogeneity; OR, odds ratios; CI, confidence interval; ins, insertion; del, deletion; F, fixed effects model; R, random effects model; Sensitivity-C, sensitivity analysis in Caucasians; Sensitivity-A, sensitivity analysis in Asians; Sensitivity-T, sensitivity in total populations. Bold values are statistically significant.

#### 3.2.3 Subgroup analysis

There were significant differences using all seven models for Caucasian and Asian subgroups. For Caribbean Hispanic subgroup, there were also significant differences using five genetic models (allelic comparison, dominant, overdominant, homozygote comparison and heterozygote comparison models), but no significant differences were found using the other two models. However, for African American subgroup, there was no significant difference using all seven models (**Table 3**).

#### 3.2.4 Stratified analysis in APOE ε4 non-carriers

Nine articles [9], [10], [12], [16], [17], [19], [20], [22], covering 10 studies, had data available to perform stratification according to *APOE* ε4 status. A subset of *APOE* ε4 non-carriers, consisting of 589 AD patients and 1301 controls, were included in the stratification analysis (**Table 4**). In *APOE* ε4 non-carriers, we found no significant association between the *APOC1* polymorphism and AD, using six genetic models (allelic comparison, recessive, dominant, overdominant, homozygote comparison, and heterozygote comparison model) (**Table 4**). Subgroup analysis by ethnicity could not be performed owing to the limited sample sizes.

**Table 4:** Summary of stratified analysis results in *APOE* ε4 non-carriers.

Genetic models	N	Groups		Heterogeneity		Mode	OR	95% CI	
		AD	controls	*P*_h_	I^2^ (%)			Lower limit	Upper limit
**Allelic comparison model**									
ins vs. del	10	589	1301	0.03	51.12	R	1.23	0.89	1.70
**Recessive model**									
ins/ins vs. ins/del+del/del	9	541	1256	0.46	0.00	F	1.85	0.91	3.80
**Dominant model**									
ins/ins+ins/del vs. del/del	9	567	1279	0.04	49.96	F	1.26	0.98	1.61
**Overdominant model**									
ins/del vs. ins/ins+del/del	10	589	1301	0.10	38.54	F	1.20	0.93	1.56
**Homozygote comparison model**									
ins/ins vs. del/del	8	410	410	0.35	10.19	F	1.95	0.93	4.12
**Heterozygote comparison model**									
ins/del vs. del/del	9	553	1259	0.06	47.19	F	1.23	0.95	1.59

Abbreviations: N, number; AD, Alzheimer's disease; *P*_h_, *P* value for heterogeneity; OR, odds ratios; CI, confidence interval; ins, insertion; del, deletion; R, random effects model; F, fixed effects model.

#### 3.2.5 Analysis of accumulation effect

To analysis the accumulation effect of *APOC1* and *APOE*, 1072 AD patients and 1907 controls were included. Despite no significant association in *APOE* ε4 carriers that did not harbor the *APOC1* insertion allele (χ^2^ = 1.11, OR = 0.86, 95% CI = 0.65–1.14, *P* = 0.29), and *APOC1* insertion allele carriers that did not harbor *APOE* ε4 (χ^2^ = 1.04, OR = 1.13, 95% CI = 0.90–1.41, *P* = 0.31), *APOE* ε4 carriers harboring the *APOC1* insertion allele, were more prevalent in AD patients than controls (χ^2^ = 119.46, OR = 2.79, 95% CI = 2.31–3.36, *P*<0.01). The PAR of *APOC1* was estimated to account for 57.49% of AD cases, while the PAR of *APOE* was 21.17%. The estimated joint PAR for AD of *APOC1* and *APOE* combined was 66.49% (**Table 5**).

**Table 5:** Analysis of accumulation effect of *APOC1* and *APOE*.

ε4 status	ins status	AD	Controls	χ^2^	OR	95% CI	*P*	PAR (%)
+	+	364	298	119.46	2.79	2.31–3.36	<0.01	66.49
+	–	82	217	1.11	0.86	0.65–1.14	0.29	21.17
–	+	143	290	1.04	1.13	0.90–1.41	0.31	57.49
–	–	483	1102	1 (reference)				

Abbreviations: ins, insertion; AD, Alzheimer's disease; OR, odds ratios; CI, confidence interval; PAR, population attributable risk.

+ means carrying at least one copy of risk allele (ε4 or ins).

– means without carrying any copies of risk allele (ε4 or ins).

#### 3.2.6 Sensitivity analyses

The results of repeated analysis showed that the corresponding pooled ORs and 95%CIs were all statistically consistent no matter in fix or random effects models. The results of repeated analysis showed that the corresponding pooled ORs and 95%CIs were all statistically similar when sequence excluding each individual study. Four articles [13], [14], [15], [23] didn’t provide the data on genotypes. Genotypes in the studies had been calculated from allele frequencies assuming HWE. Therefore, sensitivity analysis was also performed by excluding the four articles [13], [14], [15], [23] at a time to test the robustness of the results. And the results of sensitivity analysis showed that the corresponding pooled ORs and 95%CIs were all statistically consistent with the originals, with only one exception of the comparison between insertion homozygote and heterozygote in Caucasians (heterogeneity: *P*_h_ = 0.02, I^2^ = 67.01%; OR = 2.01, 95%CI = 0.37–4.65) (**Table 3**).

#### 3.2.7 Publication bias

**Figure 3:**
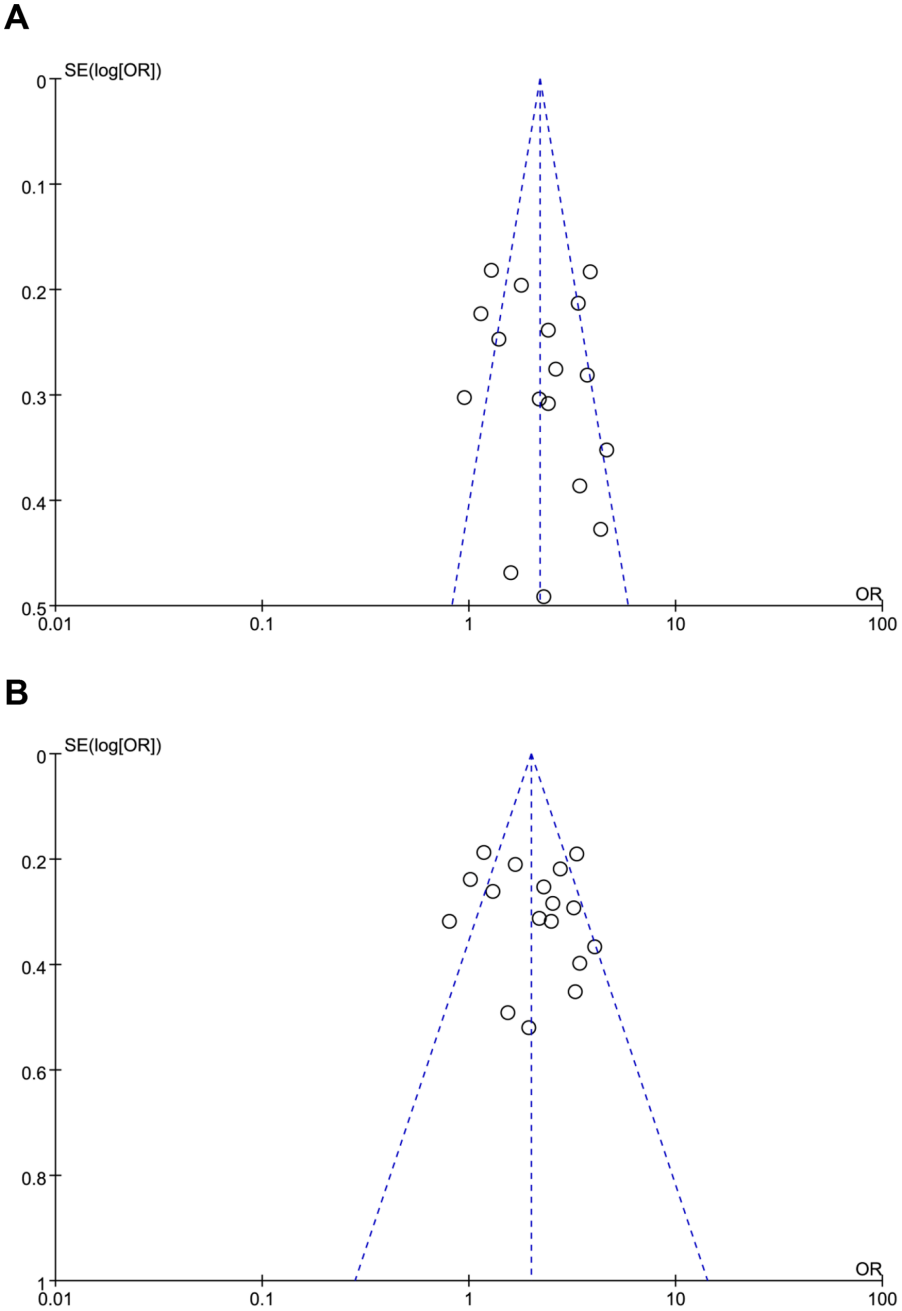
Funnel plots of *APOC1* rs11568822 polymorphism in dominant model and heterozygote comparison model. The shapes of the funnel plots revealed a degree of asymmetry visually which indicated publication bias may exist. OR: odds ratio; Log [OR]: natural logarithm of OR; SE: standard error; SE (Log [OR]): standard error of Log [OR]. Each point represents a separate study for the indicated association. (A) Dominant model. (B) Heterozygote comparison model.

The funnel plots were conducted to access the publication bias in all seven models by visual inspection. Although the shapes of the funnel plots displayed symmetry in five models (allelic comparison, recessive, overdominant, homozygote comparison models, and comparison between insertion homozygote and heterozygote), funnel plots displayed asymmetry in the other two models (dominant and heterozygote comparison models) (**Figure 3** **)**. The Egger's linear regression test was also carried out in all seven models to test statistical evidence of funnel plot asymmetry. The *P* values of Egger's linear regression test were greater than or equal to 0.10 in all seven models (**Table S1**).

## Discussion

To examine the association between the *APOC1* CGTT insertion/deletion polymorphism (rs11568822) and AD risk, stage 1 of our case-control study included 79 AD patients and 156 controls. However, the variation of *APOC1* showed no association with AD in stage 1 of our study. This result should be interpreted with caution due to the small sample size. To increase the sample size and reduce type I errors, and also test our study based on published evidence, in stage 2 we conducted a meta-analysis to further explore the association between the *APOC1* rs11568822 polymorphism and AD risk. Our meta-analysis included 2092 AD patients and 2685 controls from 15 articles (17 studies in total). We found a significant association between the *APOC1* rs11568822 polymorphism and increased AD risk in the population worldwide. Subgroup analysis by ethnic group found the *APOC1* rs11568822 polymorphism is associated with increased AD risk in Caucasians, Asians and Caribbean Hispanics, but not African Americans. Notably, the sample sizes for the Caribbean Hispanic and African American populations were limited owing to the inclusion of only two ethnic groups in one study [12]. Thus, interpretation of the results in these two ethnic groups should be treated with caution. Small sample sizes may account for either the positive result in Caribbean Hispanics or negative result in African Americans. In addition, genetic diversity among ethnic groups and their living environments may also account for differing results in different ethnic groups.

Our meta-analysis suggests the *APOC1* insertion allele is a potential AD risk allele in the population worldwide. AD risk was increased in individuals with one (ins/del, OR 1.78) or two (ins/ins, OR 3.40) copies of the insertion allele, compared with individuals with a del/del genotype (**Table 2**). AD risk was also significantly increased in individuals with two copies of the insertion allele (ins/ins, OR 1.79), compared with individuals with one copy (ins/del). Through subgroup analysis by ethnic group, we observed this gene dose-dependent effect in Caucasians, Asians and Caribbean Hispanics. In addition, the results of sensitivity analyses showed that the analyzed models, each include study, and the missing genotype data in four articles [13], [14], [15], [23] rarely affected the overall effects. The results are robust and reliable in the meta-analysis.

Using a homozygote comparison model to examine ethnicity in our meta-analysis, association between the *APOC1* insertion allele and AD risk was weaker among Asians (ins/ins, OR 2.66) and Caribbean Hispanics (ins/ins, OR 2.43), compared with Caucasians (ins/ins, OR 5.46). A similar risk effect trend was supported by the other six models (**Table 2**). Interestingly, *APOE* ε4 also appears to show this ethnically distinct pattern in AD association. The *APOE* ε4-AD association is weaker among Hispanics and African Americans, compared with Caucasian individuals [29]. As our study, many studies suggest *APOC1* alleles are not independently inherited, but often inherited in conjunction with *APOE* alleles [9], [10], [16]. *APOC1* (along with *APOE* alleles), also exhibits an ethnically distinct linkage disequilibrium pattern. The frequency of the *APOC1* insertion allele combined with *APOE* ε4 is 0.85 in European-Americans but only 0.55 in African-Americans, whereas the frequency of the *APOC1* insertion allele with *APOE* ε3 is 0.02 in European-Americans and 0.08 in African-Americans [10]. Therefore, an ethnically distinct *APOC1* pattern in association with AD risk may reflect *APOC1* linkage disequilibrium with *APOE*. There may, however, be an alternative explanation for *APOC1* ethnicity associated effects in AD. Dietary habits are likely to have an immediate impact on ethnic association of the *APOC1* polymorphism with AD risk. Epidemiological studies have shown that the Western diet includes excessive cholesterol and carbohydrate intake, and is associated with AD risk [30]. For example, cholesterol levels are greatly increased in African Americans compared with the Yoruba population, and similarly, mean serum cholesterol levels in Western populations are much higher than those found in Chinese individuals residing in China, likely reflecting dietary differences [30]. Dietary lipids are considered a major risk factor in AD development in many cross-cultural epidemiological studies [30]. Thus, different dietary habits may be a confounding factor in our results. Alternatively, the negative finding in African Americans may simply reflect the relatively small power in this study [12].

The association between the *APOC1* rs11568822 polymorphism and AD risk is in accordance with functional research. ApoC1, encoded by the *APOC1* gene, is predominantly expressed in the liver, but substantial ApoC1 expression has also been detected in brain. The CGTT insertion polymorphism in the *APOC1* promoter region, leads to a highly significant, 1.5-fold increase in expression [31]. Interestingly, in neuritic plaques of AD brain, ApoC1 was found to colocalize with Aβ and ApoE [31]. ApoE plays a key role in facilitating Aβ clearance from the brain. Supporting evidence reported that ApoC1 interferes with ApoE-mediated receptor binding, potentially inhibiting Aβ clearance and leading to Aβ deposition [10], [31]. With regards brain cognition, the hippocampus is an important cerebral structure, particularly involved in memory function. Compared with ApoC1 protein levels in frontal cortex, nearly twice are present in the hippocampus, potentially playing a crucial role in accelerated development of AD pathology [13]. Furthermore, in humans, the *APOC1* insertion allele is associated with hippocampal volume loss. The effect of *APOC1* on hippocampal volume is even more robust than the *APOE* polymorphism [32]. It is widely accepted that soluble Aβ oligomer is involved in neuronal apoptosis and impaired cognitive functions. ApoC1 exacerbates soluble Aβ oligomer-induced neuronal cell death *in vitro* [31], possibly accounting for excessive atrophy in the hippocampus and frontal cortex in AD patients. Expression of the human ins/del *APOC1* genotype in transgenic mouse brain, causes impaired hippocampal-dependent learning and memory functions [31]. Intriguingly, *APOC1* knock-out mice also display completely impaired hippocampal-dependent memory functions [33]. These two *in vivo* studies suggest an important, bell-shaped, gene-dose-dependent role for *APOC1* in specific cognitive functions. And there is little doubt that ApoC1 plays a critical and complex role in central nervous system homeostasis, since either the overexpression or absence of ApoC1 in mice impairs memory. In addition, functional research reveals apparently complex and elusive interaction between *APOC1* and *APOE*. In *APOE* ε4 individuals harboring the *APOC1* insertion allele, ApoC1 mRNA levels are strikingly lower with AD, but ApoC1 protein levels in AD were significantly higher [13]. Overall, our meta-analysis mentioned above and the functional studies lead to a hypothesis that the *APOC1* insertion allele, either alone or in combination with *APOE* ε4, is a risk factor for AD development.

In our study we attempted to distinguish between *APOC1* and *APOE* by stratification, but in AD, the *APOC1* variant with *APOE* ε4 non-carriers was not observed to be positively associated with AD risk. *APOC1* is located on chromosome 19 in the same cluster, and in close proximity, to *APOE*, therefore *APOC1* alleles are not independently inherited but rather in strict linkage disequilibrium with *APOE* alleles [9], [10], [12], [13], [14], [16], [21]. Both *APOC1* and *APOE* are in linkage as a “block”, transmitted from the parent to offspring, and therefore we should consider the accumulation effect of both of them. The joint PAR suggests that carrying both *APOE* ε4 and the *APOC*1 insertion allele, accounts for a 66.49% increase in AD risk (**Table 5**). The accumulation effect analysis indicated that the *APOC1* insertion allele combined with *APOE* ε4 serves as a risk factor for developing AD, but is no longer associated with AD susceptibility without *APOE* ε4. And it is noteworthy that many *APOC1* functional studies related to AD pathogenesis suggest an *APOC1* effect on AD development [10], [31]. Our results may be useful for identifying the true susceptibility of *APOC1* variance to AD. Another potential explanation is that we need to re-assess the *APOE* ε4 association with AD. Surprisingly, we found *APOE* ε4 was no longer associated with AD risk without the *APOC1* insertion allele. This may be due to reduced statistical power as a result of the small sample size in the accumulation effect analysis. Larger sample sizes are needed to review the interactions between *APOC1* and *APOE*, and to test if *APOE* ε4 is an independent risk factor for AD without the *APOC1* insertion allele.

Our meta-analysis pooled all available studies, increasing its statistical power. However, there are important limitations to our approach. First, substantial heterogeneity between the studies may affect reliability of our conclusions. Second, although we attempted to contact the original investigators, we were unable to obtain crucial missing genotype data in certain studies, and again this may introduce bias into our meta-analysis. Third, although Egger's linear regression tests showed no publication bias, the funnel plots of certain genetic models appeared to show a degree of asymmetry which indicated publication bias may exist in the meta-analysis. Despite systematically searching to identify eligible studies, there is still a possibility that some eligible, but unpublished studies, or studies published in languages other than English and Chinese, are not included. Fourth, several published studies didn’t provide any HWE information. Deviation from HWE may indicate the presence of genotyping errors, population stratification bias or selection bias. Fifth, a large body of evidence suggests the *APOC1* insertion allele is in linkage disequilibrium with *APOE* ε4. However, association in our meta-analysis was at the level of the allele and genotype, but not haplotype. Sixth, effects of other confounding risk factors, such as age of AD patients at onset, gender, and level of education, were not investigated in the association between *APOC1* and AD risk. Potential gene–gene and gene–environment interactions should also be taken into account when elucidating clinically important AD risk factors. Finally, the total number of participants in our meta-analysis is still relatively small, especially for the African American and Caribbean Hispanic populations, suggesting there may be inadequate power to detect the real association.

## Conclusion

Our study suggests that the *APOC1* insertion mutation, in combination with *APOE* ε4, serves as a potential risk factor for developing AD. Individuals carrying both *APOE* ε4 and the *APOC1* insertion allele had an approximately 66.49% increased risk of AD. There is substantial heterogeneity and limited sample sizes in *APOC1* association studies, underscoring the need for well-designed studies with larger sample sizes, to further examine the real *APOC1* effect with genetic networks, environmental factors, individual biological characteristics and their mutual interactions, especially in African American and Caribbean Hispanic populations. Moreover, it is important to consider gene–gene and gene–environment interactions when re-assessing the *APOE* association with AD.

## Supporting Information

Figure S1 **Electropherogram result of different** ***APOC1*** **genotypes.** M1: pBR322/MspI DNA Marker; A, C, D: del/del genotype; B: ins/del genotype; E, F: ins/ins genotype; M2: SD011 DNA Marker.
(TIF)

Table S1 **Egger's linear regression test for publication bias in different genetic models.**
(DOCX)

Checklist S1 **PRISMA Checklist.**
(DOC)
