# Supplementary figures and images for: Association between APOC1 Polymorphism and Alzheimer’s Disease: A Case-Control Study and Meta-Analysis
Source: PLoS One. 2014 Jan 31;9(1):e87017. doi: 10.1371/journal.pone.0087017 (PMC3909044; doi:10.1371/journal.pone.0087017)

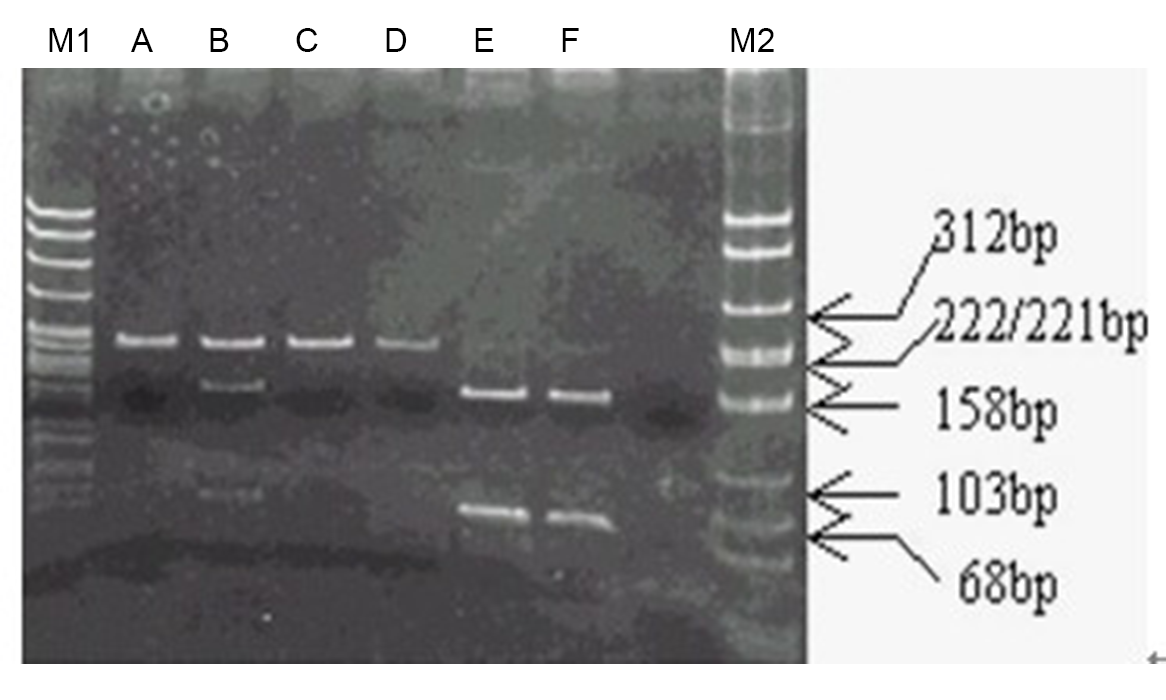

Supplement: Figure S1 — Electropherogram result of different APOC1 genotypes. M1: pBR322/MspI DNA Marker; A, C, D: del/del genotype; B: ins/del genotype; E, F: ins/ins genotype; M2: SD011 DNA Marker. (TIF) [file pone.0087017.s001.tif]
