# Supplementary material for: Association between APOC1 Polymorphism and Alzheimer’s Disease: A Case-Control Study and Meta-Analysis
Source: PLoS One. 2014 Jan 31;9(1):e87017. doi: 10.1371/journal.pone.0087017 (PMC3909044; doi:10.1371/journal.pone.0087017)
Supplement: Table S1 — Egger's linear regression test for publication bias in different genetic models. (DOCX) [file pone.0087017.s002.docx]

**Table S1** Egger's linear regression test for publication bias in different genetic models.

| Genetic models | Standard error | t | P-value | 95% CI of intercept |
| --- | --- | --- | --- | --- |
| Allelic comparison model | 1.55 | 0.61 | 0.55 | -2.36， 4.26 |
| Recessive model | 0.72 | 1.74 | 0.10 | -0.28， 2.77 |
| Dominant model | 2.10 | 1.58 | 0.14 | -1.36， 8.01 |
| Overdominant model | 1.32 | 0.88 | 0.39 | -1.65， 3.98 |
| Homozygote comparison model | 0.87 | 1.40 | 0.18 | -0.64， 3.07 |
| Heterozygote comparison model | 2.00 | 1.55 | 0.15 | -1.35， 7.56 |
| Homozygote vs. heterozygote | 0.64 | 1.27 | 0.22 | -0.56, 2.20 |
| Abbreviations: CI, confidence interval. | |  |  |  |
